# GSDME enhances Cisplatin sensitivity to regress non-small cell lung carcinoma by mediating pyroptosis to trigger antitumor immunocyte infiltration

**DOI:** 10.1038/s41392-020-00274-9

**Published:** 2020-08-24

**Authors:** Zhouyangfan Peng, Pengfei Wang, Wei Song, Qingmei Yao, Yinjia Li, Linglong Liu, Yapei Li, Sufang Zhou

**Affiliations:** 1 Department of Hematology and Critical Care Medicine, The Third Xiangya Hospital, Central South University, Changsha, 410013 Hunan China; 2 Department of Biochemistry and Molecular Biology, School of Basic Medical Sciences, Guangxi Medical University, Nanning, 530021 Guangxi China; 3 Health Management Center, The Third Xiangya Hospital, Central South University, Changsha, 410013 Hunan China

**Keywords:** Tumour immunology, Lung cancer

**Dear Editor,**

Non-small cell lung cancer (NSCLC) is the most common type of lung cancers. Since the majority of NSCLC patients are diagnosed at the advanced stage with a dramatic impact on the prognosis, chemotherapy is still the mainstay of treatment. Cisplatin (CDDP) remains one of the most widely used first-line drugs in the therapy of NSCLC, based on the clinical data that the poor outcomes of untreated NSCLC patients (5% overall survival at 1 year) have shown improvements in natural history of disease after CDDP-based chemotherapy (15% overall survival at 1 year).^1^ Nevertheless, Cisplatin has strong toxic side effects and many NSCLC patients treated with CDDP are easy to occur drug insensitivity, near-to-invariably leading to relapse and therapeutic failure. Therefore, finding novel target molecules which can regulate CDDP innate sensitivity is vital to improve clinical efficacy. For decades, a long-held consensus is that caspases-3-induced apoptosis is the major pattern for antitumor role of CDDP. The CDDP can promote cancer cell elimination via rewiring pro-proliferative signaling to pro-apoptotic pathway. However, apoptosis resistance readily occurs after CDDP treatment and it is of great significance to explore whether other types of programmed cell death can effectively heighten innate CDDP sensitivity.

Pyroptosis, as a faster and severer type of programmed cell death than apoptosis, has long been thought to be mediated by gasdermin family member gasdermin D (GSDMD). Recent studies have found that gasdermin E (GSDME) can switch apoptosis to pyroptosis in GSDME-high expression cells by targeting to caspase-3 to competitively inhibit the combination of caspase-3 and apoptotic substrates.^2^ It has also been proved that molecular targeted antitumor agents can induce GSDME-mediated pyroptosis.^3^ But whether GSDME is associated with the clinical CDDP therapeutic effect still need to be further elucidated. Although GSDMD can trigger inflammatory factors release through mediating extensive pore forming,^4^ the role of GSDME-mediated inflammatory factors releases, especially in enhancing CDDP sensitivity, is still unclear.

We have performed immunohistochemical (IHC) of a lung cancer tissue microarray (TMA) for GSDME and quantified GSDME expression in paired normal lung and lung carcinoma tissues from 90 NSCLC patients. We have found that GSDME expression significantly reduces in the majority of lung cancer tissues compared with paired normal lung tissues (Fig. 1a). In spite of decreased GSDME levels have not been related to the tumor size, clinical stage, age, and tumor recurrence rate in NSCLC patients (Supplementary Fig. S1a–f), we have unexpectedly found that patients with lower GSDME expression in tumor tissues have shorter survival time and higher mortality rate after platinum treatment (Fig. 1b). The T-cell infiltration degree of lung cancer tumor is higher in platinum treatment patients with higher GSDME expression in tumor tissues (Supplementary Fig. S2a, b). Those results suggest that GSDME expression may play a key role in innate platinum drugs sensitivity.

**Fig. 1:**
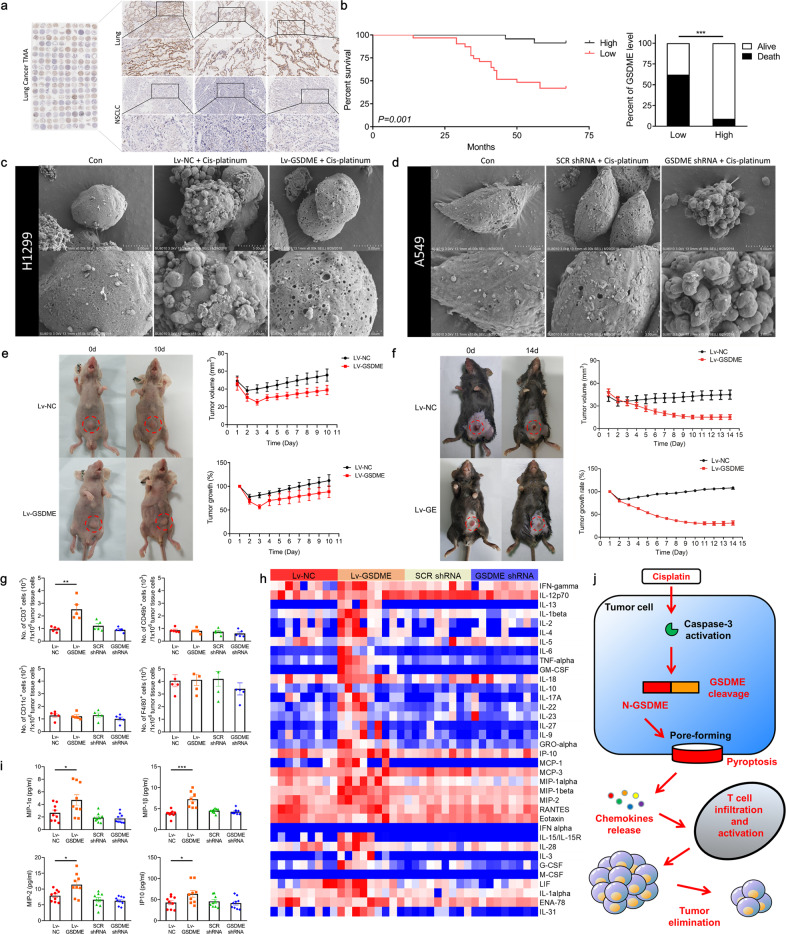
The immune-regulation role of GSDME enhances CDDP sensitivity to regress NSCLC. **a** Immunohistochemical (IHC) staining of GSDME in a lung cancer tissue microarray (TMA) that contained 90 specimens, GSDME expression was analyzed in paired NSCLC lung cancer and its surrounding tissue, representative images of IHC staining were shown. **b** The difference of survival time and death number in NSCLC patients with low- and high-GSDME tumors that accepted chemotherapy with platinum. **c** Representative scanning electronic micrographs of H1299 cells with negative control or GSDME-overexpression lentivirus and then treated with cisplatin. **d** Representative scanning electronic micrographs of A549 cells with scrambled shRNA or GSDME shRNA lentivirus and then treated with cisplatin. **e** The representative image and statistical data in nude mice bearing LLC xenografts contained with negative control or GSDME-overexpressing lentivirus. **f** The representative image and statistical data in C57 mice bearing LLC xenografts contained with negative control or GSDME-overexpressing lentivirus. **g** CD3^+^, CD49b^+^, CD11c^+^, and F4/80^+^ cell number were analyzed by flow cytometry in mice with indicated LLC tumors treated with cisplatin. **h** Heatmap of cytokines in mice blood with indicated LLC tumors. **i** The statistical data of MIP-1α, MIP-1β, MIP-2, and IP-10 levels in mice blood. **j** A schematic representation of proposed mechanism for the role of GSDME in NSCLC

In order to get insights into the relationship between GSDME expression and CDDP sensitivity, we have selected four types of lung cancer cells which express different levels of GSDME and found that GSDME-high expressing cells are more sensitive to CDDP (Supplementary Fig. S3a, b). Contrary to the apoptotic response in control H1299 cells with natural GSDME-low expression, overexpressing GSDME by lentivirus in H1299 cells switch apoptotic death to pyroptotic death after incubation with CDDP (Fig. 1c, Supplementary Fig. S4a–d). The GSDME-high expression A549 cells have shown pyroptotic death after CDDP treatment, but occurred apoptosis in GSDME-silencing A549 cells (Fig. 1d, Supplementary Fig. S4e–h).

We have further detected the role of GSDME in CDDP-induced cancer cell elimination. Compared with negative control cells, GSDME-overexpressed A549 and H1299 cells have both exhibited less cell viability after CDDP treatment (Supplementary Fig. S5a–d). Conversely, GSDME-silenced cancer cells have shown decreased CDDP response (Supplementary Fig. S5e–h). Similar results have also been obtained in clonogenic cell survival assays. The clonogenic potential of A549 and H1299 cells has been inhibited by GSDME overexpression after exposure to CDDP (Supplementary Fig. S5i, j). But the clonogenic potential of GSDME-silencing cells of A549 and H1299 has been barely affected by exposure to CDDP (Supplementary Fig. S5k, l). These results suggest that GSDME-mediated pyroptosis increases the sensitivity of CDDP to NSCLC cells.

We have next established xenograft model in nude mice via injecting LLC, A549, or H1299 cells, which stably overexpress or silence GSDME. Contrast to normal control cells, GSDME-overexpressed cells have exhibited enhanced drug sensitivity after CDDP treatment. Although the therapeutic index has increased transiently and then reduced gradually, the therapeutic index of CDDP in overexpressed-GSDME tumor is always higher than the control group (Fig. 1e, Supplementary Fig. S6a, b). The cancer cells with genetic GSDME silencing can resist CDDP-induced tumor growth inhibition (Supplementary Fig. S6c, d). Those results suggest that GSDME-mediated cell death can only inhibit tumor growth, not further trigger regression of tumor.

Since in clinical studies we have found that the high expression of GSDME can promote T cells infiltration in CDDP-treatment patient tumor tissues, we have speculated that this may be a more important way for GSDME to exert antitumor therapeutic effects. While nude mice are thymic-deficiency, we have hypothesized that GSDME may provide a better antitumor effect of CDDP treatment in C57 mice due to its normal immune function. Mouse-derived LLC lung cancer cells have been injected to C57 to establish xenograft. As nude mice, the growth rate of GSDME-silenced LLC tumor has been increased after CDDP treatment (Supplementary Fig. S7a). But it is beyond our expectation that the prominently reduction of tumor volume has been found in GSDME-overexpressing LLC xenograft in C57 mice after CDDP treatment (Fig. 1f), indicating that GSDME probably involve in the antitumor therapy via immune regulation.

According to our hypothesis that GSDME may promote immunocyte infiltration through mediating pyroptotic pore forming to regulate immune factor release, we have performed flow cytometry and found that GSDME overexpression increases the number of tumor-infiltrating CD3^+^ T cells (Fig. 1g). We have also analyzed the number of other immune cells in tumor tissues, and found that GSDME overexpression do not promote infiltration of other immune cells, including B cells, NK cells, dendritic cells, and macrophages (Fig. 1g). Meanwhile, we have used cytometric bead array (CBA), which can identify 36 kinds of cytokines, to detect the cytokine release effect of GSDME on these xenograft models (Fig. 1h, Supplementary Fig. S8a). Our results have shown that overexpression of GSDME in LLC xenograft induces increased MIP-1α, MIP-1β, MIP-2, and IP-10 levels both in the tumor tissue and blood after CDDP treatment, those chemokines have been shown to play an important role in T-cell recruitment (Fig. 1i, Supplementary Fig. S8b). Those data suggest that GSDME-mediated pyroptosis can promote T-cell recruitment by releasing the related cytokines. The TNF-α and IFN-γ as important antitumor molecules are mainly released by activated T cells.^5^ In our results, the level of both cytokines is significantly increased in tumor tissue and blood, suggesting that GSDME may induce T-cell activation in the process of promoting T-cell infiltration into tumor tissues (Supplementary Fig. S9a, b).

In summary, this study has expanded the conventional view regarding CDDP only induced a sole death route by caspase-3-mediated apoptosis. We have first provided several lines of evidence to prove that GSDME-mediated pyroptosis provides a novel antitumor role of CDDP treatment by releasing chemokines to recruit T cell in tumor tissue. The detection of GSDME expression before cisplatin treatment can effectively differentiate the CDDP sensitivity in NSCLC patients. The discovery of immunological regulation effect of GSDME in lung cancer also provides a new potential target for the development of lung cancer immunotherapy.

## Supplementary information

Supplementary Materials

Supplementary table
