# Supplementary material for: GSDME enhances Cisplatin sensitivity to regress non-small cell lung carcinoma by mediating pyroptosis to trigger antitumor immunocyte infiltration
Source: Signal Transduct Target Ther. 2020 Aug 24;5:159. doi: 10.1038/s41392-020-00274-9 (PMC7445264; doi:10.1038/s41392-020-00274-9)
Supplement: Supplementary file 1 — Supplementary Materials [file 41392_2020_274_MOESM1_ESM.docx]

Supplementary Materials for

**GSDME enhances Cisplatin sensitivity to regress** **non–small cell lung carcinoma by mediating pyroptosis** **to trigger antitumor immunocyte infiltration**

**Author information**

Zhouyangfan Peng^3†^, Pengfei Wang^1†^, Wei Song^1†^, Qingmei Yao^1^, Yinjia Li^1^, Linglong Liu^1^, Yapei Li^2*^ & Sufang Zhou^1,4*^

^1^School of Preclinical Medicine, Guangxi Medical University, Nanning, Guangxi, China.

^2^Department of Health Management, the Third Xiangya Hospital, Central South University, Changsha, China.

^3^Department of Hematology, the Third Xiangya Hospital, Central South University, Changsha, China.

^4^National Center for International Research of Biological Targeting Diagnosis and Therapy, Guangxi Medical University, Nanning, Guangxi, China.

^†^P-ZYF, W-PF and S-W equally contributed to this work.

^*^Correspondence: Yapei Li & Sufang Zhou

E-mail: [Sunshine0726@csu.edu.cn](mailto:Sunshine0726@csu.edu.cn); [zsf200000@163.com](mailto:zsf200000@163.com);

**This PDF file includes:**

Materials and Methods

Fig. S1 to S9

**Materials and Methods**

**Patient samples**

The study was approved by the Ethics Committees of Guangxi medicine university. Written informed consent was acquired from all patients in this study. Fresh-frozen tumor samples were obtained from patients during surgery, and formalin-fixed and paraffin-embedded (FFPE) sections were collected during pathological examination. Details of patient information were shown in Supplementary Tables S1.

**Cell culture and reagents**

Cell lines including 293T, A549, H1299, H226, H23 and LLC were obtained from Cellbank of Chinese Academy of Sciences. Cells were cultured in RPMI-1640 (Gibco) medium, supplemented with 10% fetal bovine serum (Gibco), 100 U/ml penicillin and 0.1 mg/ml streptomycin at 37°C and 5% CO_2_ Cisplatin (Selleck Chemicals) was reconstituted in normal saline at a stock concentration of 10 mM and used to incubate cells at an indicated concentration. LDH assays were performed using the CyQUANT LDH cytotoxicity assay (Thermo Fisher) according to the manufacturer’s instructions.

**Lentivirus transfections**

To establish stable overexpressed or knockdown of GSDME expression cells, cells (A549, H1299 and LLC) were seeded onto 6-well plates at a density of 2 × 10^5^ cells / well and transfected with lentivirus (MOI = 20) containing GSDME cDNA or shRNA and non-targeting cDNA or shRNA with GFP (negative control) to cell. Then cells were used for the following treatment including in vitro and in vivo experiments.

**Flow Cytometry**

Flow cytometry analyses was used to determine cell death, A549 cells and H1299 cells were transfected with lentivirus and then treated with CDDP as indicated. Cells were collected, washed with PBS, resuspended in 100μl binding buffer and stained using 5 μl Annexin V-FITC/PE (BD sciences) by following the manufacturer’s instruction. Stained cells were analyzed on the BD FACSAria III flow cytometer and data were processed by the FlowJo software.

**Scanning electron microscopy**

Cells grown on glass coverslips were washed with PBS, and then fixed with 3% glutaraldehyde overnight at 4 °C, followed by rinsing with PBS for three times. Samples were dehydrated through a graded series of ethanol (50, 70, 80, 95 and 100%) and dried by Critical Point Dryer CPD 300 (Leica). Dried specimens were sputter coated with gold-palladium by Super Cool Sputter Coater SCD050 (Leica) and imaged with a scanning electron microscope S3400N-II (Hitachi) operating at 10 kV.

**Clonogenic cell survival assays**

Clonogenic survival was evaluated in colony formation assays, cell suspensions were seeded into 6-well plates at different concentrations (5×10^2^ per well), after adhere overnight, cells were treated with CDDP or vehicle for 24 hours, and then cultured in drug-free medium for the following 10 days. Eventually, colonies were fixed with formalin and stained with 0.25% (w/v) crystal violet and colonies with more than 30 cells were included in the quantification. The surviving fraction (SF) was calculated according to the formula: SF ¼ (number of colonies formed/number of cells seeded)

**CCK8 assays**

Cells viability was assessed by using the Cell Counting Kit-8 (CCK8) assay (BioRad) according to the manufacturer's recommendations. Briefly, 8 × 10^3^ cells / well were seeded into 96-well plates and allowed to adhere overnight. Indicated cells were treated with CDDP or vehicle. Subsequently, CCK8 solution was added to each well and incubated for 2 h at 37°C. Absorbance was measured using a microplate reader (BioRad) at a wavelength of 450 nm.

**Tumor xenograft models**

All animal care and experiments were approved by the Ethics Committees of the Guangxi Medical University. The related procedures were in accordance with the Association for Assessment and Accreditation of Laboratory Animal Care guidelines and conformed to the Animal Protection Act of China. Indicated A549, H1299 and LLC cancer cells (5 × 10^6^ cells suspended in 100 μl PBS) were subcutaneously implanted in the two sides of abdomens of BALB/c nude mice (4 weeks old, 15 ~ 18g weight, half of male and half of female). Animals were treated with cisplatin (8 mg/kg on day 0) or vehicle control (0.9% physiological saline), while tumor sizes reached 200 ~ 400 mm^3^.

To evaluate the function of immune system during the treatment, C57BL/6 mice (6 weeks old, 18 ~ 22g weight, half of male and half of female) were also used as xenograft models. 2 × 10^6^ of LLC cells were subcutaneous injected into abdomen of the mice. When tumor sizes reached approximately 200 ~ 400 mm^3^, 8 mg/kg cisplatin was administered at day 0. Tumor growth and body weight were measured at each day. Tumor volumes were measured with a caliper and calculated as length × width^2^ × 0.5([48](#_ENREF_48)). All mice were sacrificed at indicated time point. Tumor tissues and blood samples were collected for further examination.

**Cytometric Bead Array**

Blood and tumor tissues were both performed cytometric bead array. Blood was extracted through cardiac puncture. After centrifugation for 10 minutes at 3,000 rpm, serum was stored at -80 °C before analysis. Separated tumor tissues were grinded by tissue grinder. The prepared tissue homogenates were used to analysis. Cytokine levels were measured using a Multiplex Luminex assay (BD sciences). Reagents for quantitative ProcartaPlex Luminex immunoassay were sourced from Affymetrix eBioscience. Cytometric Bead Array (Thermo Fisher) were used according to the manufacturer’s instructions. And results were read on the Bio-Plex 200 instrument.

**Western blot**

Western blot was used to analyze protein expression as described previously. In brief, Cells or tissue samples were collected and lysed in RIPA buffer containing proteinase inhibitors (Roche) and phosphatase inhibitors (Roche). The protein extracts (30 μg/lane) were separated on SDS-PAGE gels, followed by electrotransfer onto polyvinylidene fluoride membranes (Sigma-Aldrich). After blocking with 5% fat-free milk for 2 h at room temperature, the membranes were incubated with primary antibodies and subsequently incubated with secondary antibody (Santa Cruz Biotechnology). Finally, bands were scanned with Bio-Rad Imager and individual band intensity was determined with software of ImageJ. Antibodies against the following proteins were used: caspase-3 (CST), cleaved caspase-3 (CST), GSDME (Abcam), β-actin (Proteintech).

**Immunohistochemistry**

Acquisition, paraffin embedding, antibody staining and pathological scoring of lung adenocarcinoma and squamous cell carcinoma samples were performed by Shanghai Outdo Biotech Company. All specimens were handed anonymously following the company’s protocol and ethical standards. Paraffin-embedded sections were immunolabeled with GSDME or CD3 antibody (Abcam). Positive staining and staining scores were determined by a pathologist in cancer cells and peri-tumoral normal tissues. GSDME expression was calculated by multiplying the percentage of positivity with staining score in each sample.

**Statistical analysis**

Statistical analysis was performed with GraphPad Prism software, and data are presented as mean ± SD. In all experiments, comparisons between two groups were based on unpaired Student’s t-test and one-way analysis of variance (ANOVA) was used to test the difference among more groups. The Kaplan-Meier method was used to compare differences in mortality rates between groups. *P*-values of <0.05 were considered statistically significant.

**
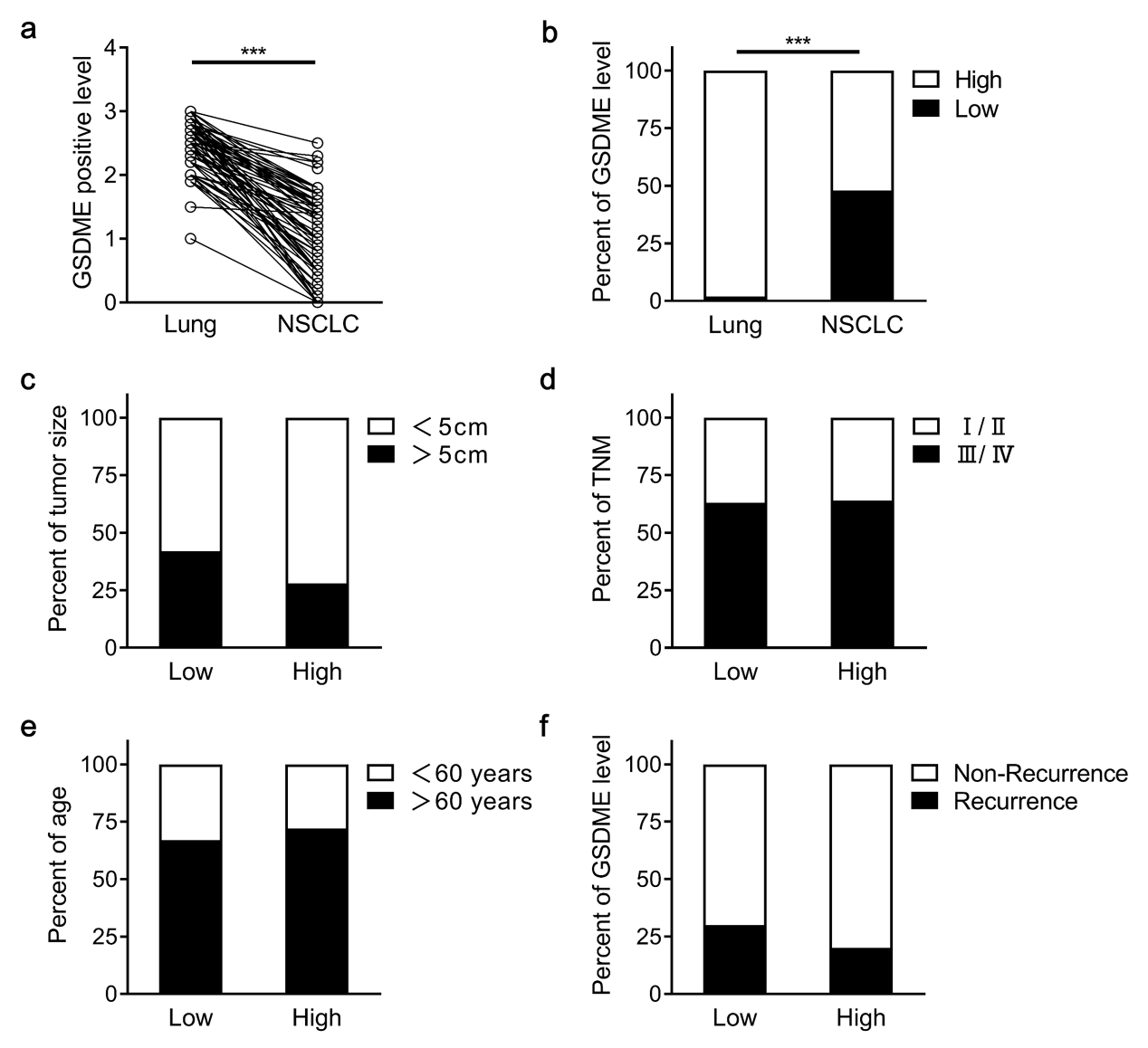
**

**Fig. S1 Expression and functional implications of GSDME in lung cancer.**

(a) GSDME positive level in the lung cancer and paired normal tissue. Statistical significance was determined by paired Student’s t-test.

(b) Quantification of GSDME expression status on the basis of IHC staining, the percentage of GSDME level in the lung cancer and its paired surrounding tissue. According to the staining score, the expression level of GSDME divided into low and high.

(c) The difference of tumor sizes in the low- and high-GSDME group.

(d) The difference of clinical TNM stage in the low- and high-GSDME group. TNM, pathologic tumor, lymph node, metastasis classification.

(e) The difference of age (>60 years and <60 years) in the low- and high-GSDME group.

(f) The difference of recurrence rate in NSCLC patients with low- and high-GSDME tumors that accepted chemotherapy with platinum.

**
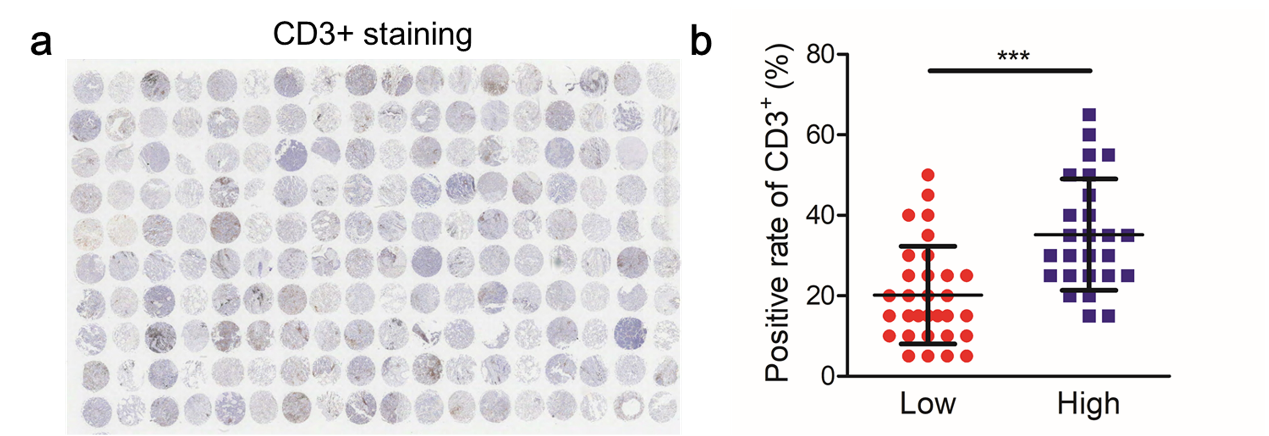
**

**Fig. S2 Expression of CD3 in lung cancer.**

(a) Immunohistochemical (IHC) staining of CD3 in a lung cancer tissue microarray (TMA) that contained 90 specimens.

(b) The difference of CD3 expression in NSCLC patients with low- and high-GSDME tumors that accepted chemotherapy with platinum.

**
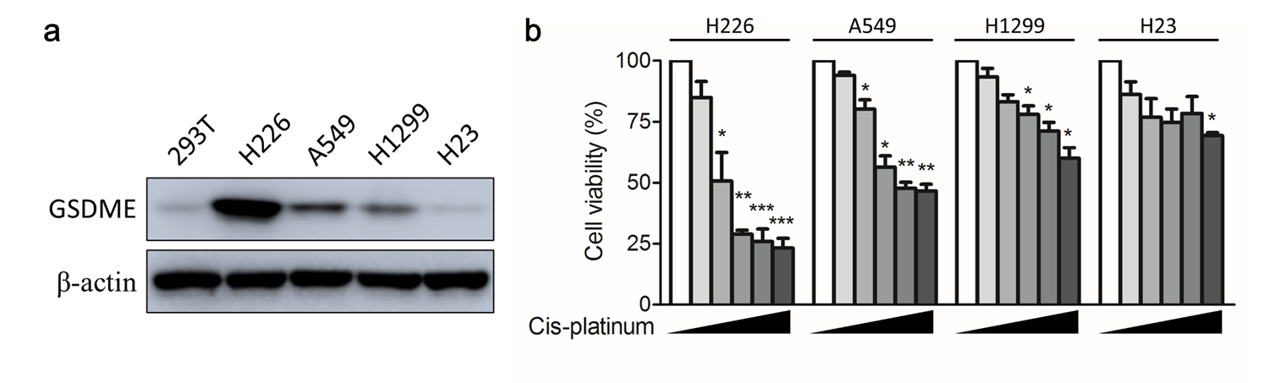
**

**Fig. S3 Cisplatin therapies activate GSDME-dependent pyroptosis in GSDME high expression cancer cells.**

(a) GSDME protein expression were analyzed by Western blotting.

(b) H226, A549, H1299 and H23 cells were treated with different doses of cisplatin for 24h, cell proliferation ability was detected by CCK8.

**
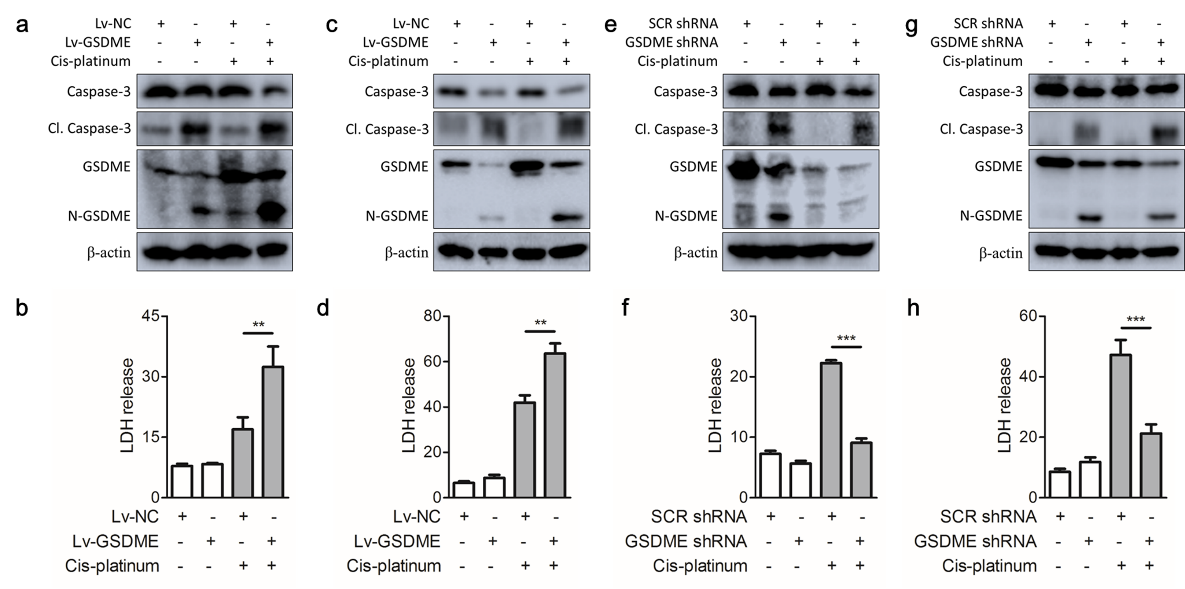
**

**Fig. S4 Cisplatin therapies activate GSDME-dependent pyroptosis in GSDME high expression cancer cells.**

(a) A549 were transfected with GSDME overexpressing lentivirus and then cells were treated with indicated dose of cisplatin, GSDME and caspases-3 expression were detected with immunoblotting.

(b) LDH release were detected in CDDP-treated A549 transfected with GSDME overexpressing lentivirus.

(c) H1299 were transfected with GSDME overexpressing lentivirus and then cells were treated with indicated dose of cisplatin, GSDME and caspases-3 expression were detected with immunoblotting.

(d) LDH release were detected in CDDP-treated H1299 transfected with GSDME overexpressing lentivirus.

(e) A549 were transfected with GSDME silencing lentivirus and then cells were treated with indicated dose of cisplatin, GSDME and caspases-3 expression were detected with immunoblotting.

(f) LDH release were detected in CDDP-treated A549 transfected with GSDME silencing lentivirus.

(g) H1299 were transfected with GSDME silencing lentivirus and then cells were treated with indicated dose of cisplatin, GSDME and caspases-3 expression were detected with immunoblotting.

(h) LDH release were detected in CDDP-treated H1299 transfected with GSDME silencing lentivirus.

All data shown are representative of at least three independent experiments.

**
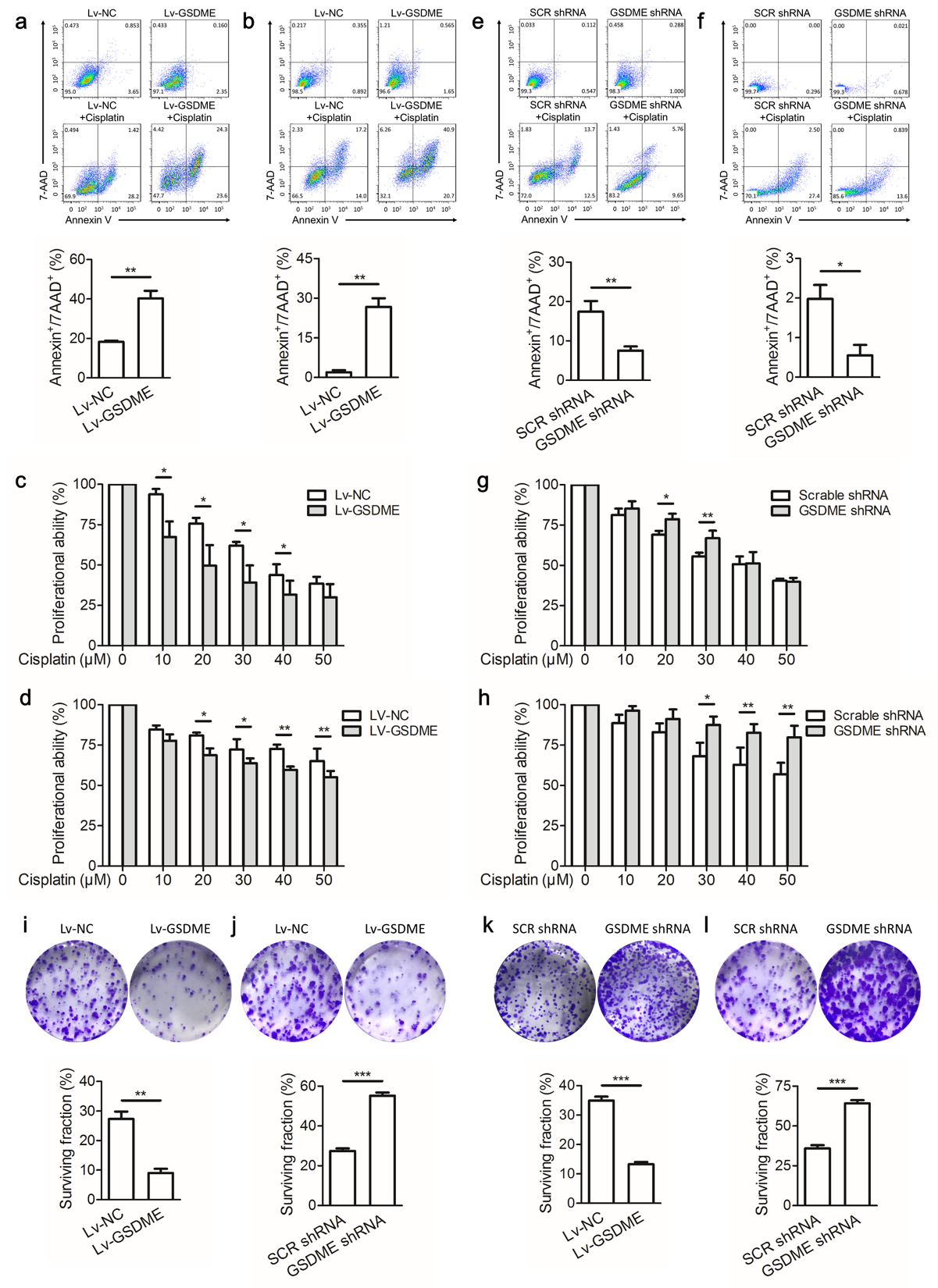
**

**Fig. S5 Cisplatin induces cancer cell elimination is depended on GSDME expression level.**

(a,b) Flow-cytometry assays for GSDME-overexpressing A549 and H1299 cells that stained by PE and 7-AAD.

(c,d) GSDME-overexpressing A549 and H1299 cells were treated with different doses of CDDP and evaluated proliferation ability by CCK8.

(e,f) Flow-cytometry assays for GSDME-silencing A549 and H1299 cells that stained by PE and 7-AAD.

(g,h) GSDME-silencing A549 and H1299 cells were treated with different doses of CDDP and evaluated proliferation ability by CCK8.

(i,j) Clone formation in GSDME-overexpressing A549 and H1299 cells with CDDP treatment.

(k,l) Clone formation in GSDME-silencing A549 and H1299 cells with CDDP treatment.

**
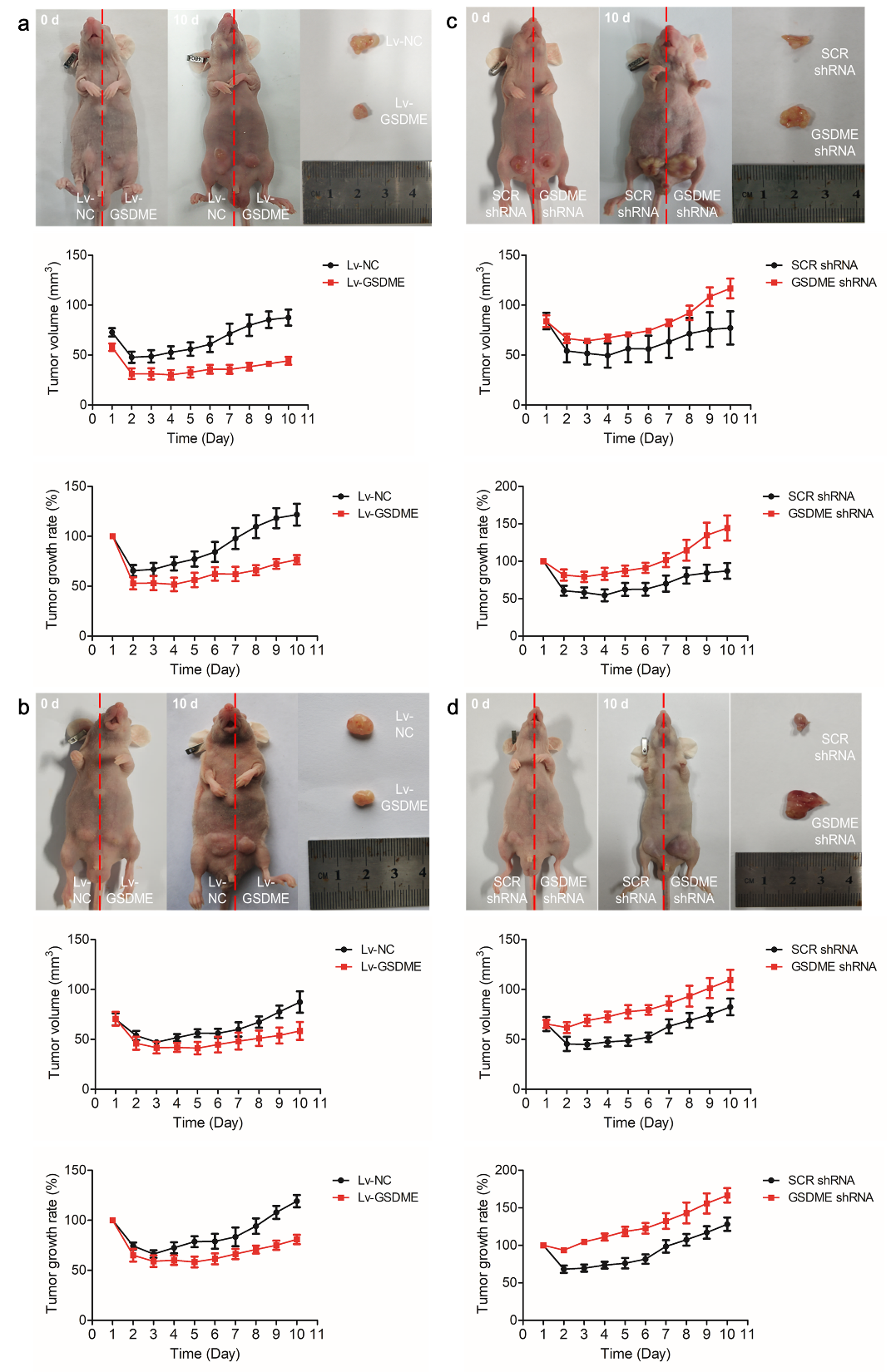
**

**Fig. S6 GSDME transiently elevates anti-tumor role of cisplatin treatment in BALB/c nude mice with A549 or H1299 xenograft models.**

(a) The representative image and statistical data in nude mice bearing A549 xenografts contained with negative control or GSDME-overexpressing lentivirus

(b) The representative image and statistical data in nude mice bearing H1299 xenografts contained with negative control or GSDME-overexpressing lentivirus

(c) The representative image and statistical data in nude mice bearing A549 xenografts contained with negative control or GSDME-silencing lentivirus

(d) The representative image and statistical data in nude mice bearing H1299 xenografts contained with negative control or GSDME-silencing lentivirus

**
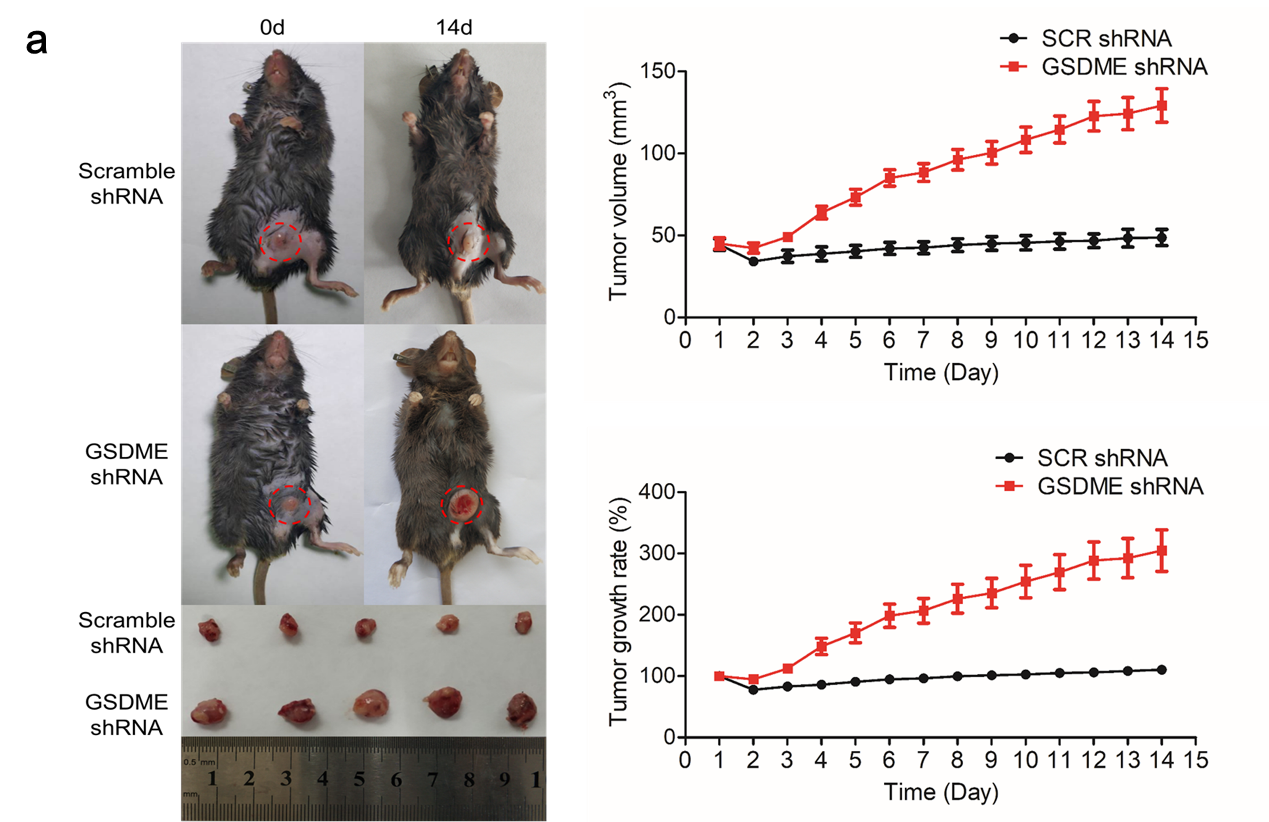
**

**Fig. S7 Silencing GSDME inhibits cisplatin treatment effect in C57 mice with LLC xenograft models.**

(a) The representative image and statistical data in C57 mice bearing LLC xenografts contained with negative control or GSDME-silencing lentivirus

**
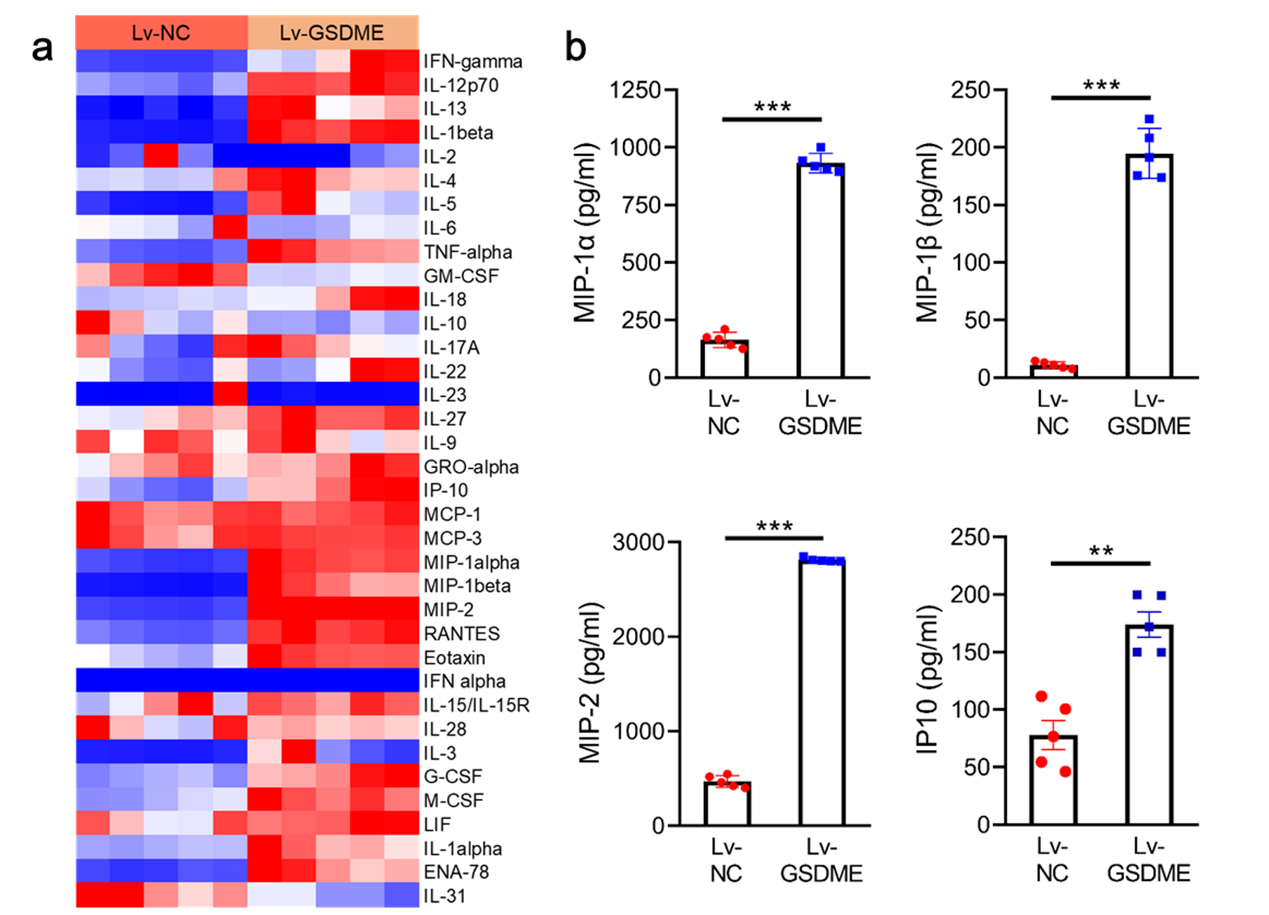
**

**Fig. S8 Cytokines level in mice tumor tissues**.

(a) Heatmap of cytokines in mice tumor tissues with indicated LLC tumors.

(b) The statistical data of MIP-1α, MIP-1β, MIP-2, IP-10 levels in mice tumor tissues.

**
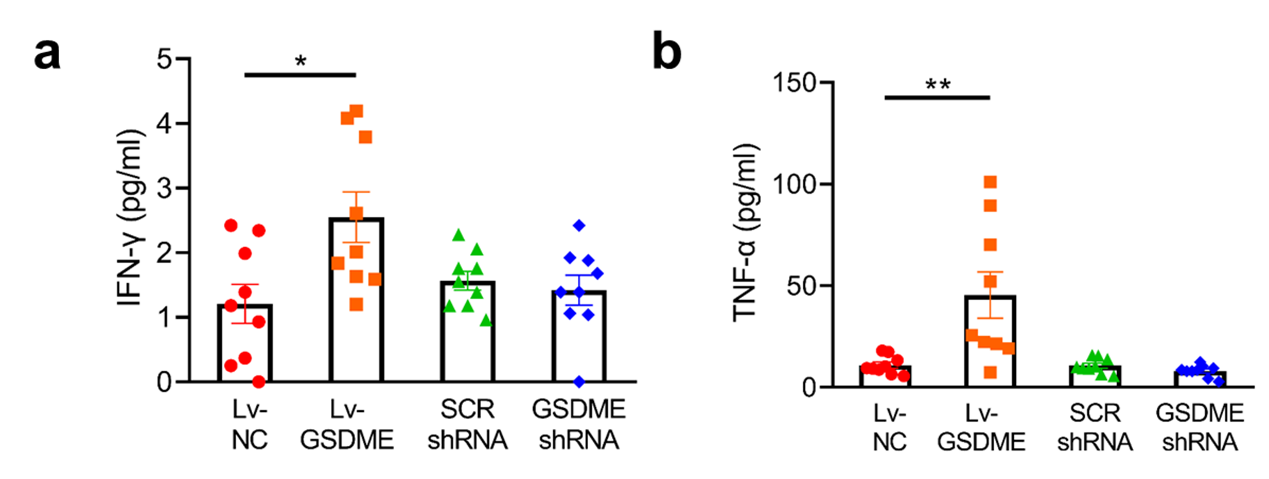
**

**Fig. S9 The level of IFN-γ and TNF-α in C57 mice blood bearing LLC xenograft**

(a) IFN-γ levels in mice blood with indicated LLC tumors.

(b) TNF-α levels in mice blood with indicated LLC tumors.
